# Principles of Physics and Meteorology

**Published:** 1848-04

**Authors:** 


### BIBLIOGRAPHICAL NOTICES.

*Principles of Physics and Meteorology.* By J. Müller, Professor of Physics at the University of Freiburg. *First American Edition. Revised and Illustrated with* 538 *Engravings on wood and two Coloured Plates.* Philadelphia. Lea & Blanchard: 1848.

At the present day it can hardly be requisite to speak of the absolute necessity for a well-educated physician to be conversant with Natural Philosophy. At every turn he is met with the need of a knowledge of its principles; and, in proportion as he is well instructed in these, *cæteris paribus*, will he be prepared to practice successfully and with advantage to himself. That this is so, we have only to reflect, for a moment, upon the human body. What is it but a piece of mechanism of the most beautiful description?

In the osseous and muscular systems the principles of the lever and of active forces are exemplified; those of hydraulics in the vascular, and of pneumatics in the respiratory system.

And then, too, the Eye. What an admirable structure! and yet in all its delicate arrangement obedient to physical laws—itself the most perfect achromatic instrument. But a copy of this is that wonderful mechanical instrument, the telescope, by means of which man looks upward amid the starry host—brings within his vision the remotest planet, and gives it a local habitation and a name.

No surgeon, thoroughly imbued with the laws of Physics, can ever be at a loss in the application of his various apparatus, to meet the continually changing requisitions of his art; without a practical knowledge of the same, he must ever be a novice and a bungler.

True it is, as Dr. Arnott observes, that there is scarcely a part of the animal body, or an action which it performs, or an accident that can befal it, or a piece of professional assistance which can be given it, that does not furnish illustration of some truth of natural philosophy.

The present work contains sections on the following subjects:

General Properties of Bodies—Equilibrium of Forces—Motion and accelerating Forces—Acoustics—Light—Magnetism—Electricity—Galvanism—Electro-Magnetism—Meteorology.

Several articles have been added to the London edition by the American publishers; *e.g.*, those on the electro-magnetic telegraph, electrotype, the steam engine, &c. &c.

*Light.* Two theories have divided the opinions of men of science concerning the phenomena of light, to wit: that of emission, and that of undulation. Both regard it as imponderable. But the theory of undulation regards light as propagated by the vibrations of a subtile matter termed ether.

"This Ether," says Müller, "fills the whole universe, since light penetrates the spaces of the heavens. This imponderable substance is not only distributed through the otherwise vacant space, separating the stars, but it penetrates all bodies, filling up the interstices occurring between the ponderable atoms. If the ether were in a state of rest throughout the whole universe, there would everywhere be darkness; but put into vibration, as it were, at one spot, the waves of light are propagated in all directions, as the vibrations of a cord are transmitted through a calm atmosphere."

This, no doubt, is now the prevailing opinion of philosophers. The great German poet and savant, Goethe, gave it the aid of his powerful mind, and aided in establishing a more reasonable theory than that of Newton.

*Diurnal Variations of Temperature.* To observe from hour to hour the variations of the thermometer is laborious, and a greater tax upon time than can well be afforded. Hence observers seek to obtain the *mean temperature* of the day.

This, according to Müller, is best done by observing the thermometer at several *similar* hours, "for instance, at 4 and 10, A.M., and at 4 and 10, P. M.; this method is, as Brewster has shown, correct to one-tenth of a degree: we likewise obtain a very useful result by making our observations at 7, A. M., at noon, and at 10, P. M., and then taking the mean of these three periods."

The distribution of heat over the surface of the earth was first distinctly brought to view by the celebrated German traveller and naturalist, Humboldt. He established for this purpose, what he called *isothermal lines*. These imaginary lines were drawn through all those places in the same hemisphere which have equal mean annual temperatures.

Such a line, however, passes through places which have by no means the same degree of latitude.

The difference of temperature between the two continents—Europe and America—on the same parallels of latitude, has always been a perplexing subject.

Though the extract is somewhat long, we venture to give it, as it embraces Müller's views on this point and tends to clear up the difficulty to some extent.

"In the northern temperate zone, south-west and north-east winds prevail. The former come from the equatorial districts, and partially bear the heat of the tropics towards colder regions: this warming influence of the south-west winds is, however, most marked in those districts which are the most exposed to south-western currents of air, and thus we see why it is that the western shores of great continents become warmer than the east coasts, and that the isothermal lines in Europe, which is actually only a peninsular prolongation of the Asiatic continent, and on the western shores of North America, ascend further to the north than in the interior of Asia and on the eastern shores of North America.

"A second cause to which Europe owes its relatively warm climate, is this: that in the equatorial region it is bounded towards the south, not by sea, but by an extensive continent, Africa, whose vast extent of desert and sand render it extremely hot when exposed to the vertical solar rays. A warm current of air rises continually from the glowing hot sandy waste, to descend again in Europe.

"Finally the current known by the name of the Gulf Stream contributes considerably to make the European climate milder. The origin of this current is to be sought for in the Gulf of Mexico, where the water is at a temperature of 87° F.

"Issuing from the gulf, between Cuba and Florida, the stream at first skirts the American shores, and then, as it comes into higher latitudes, turns with decreasing temperature eastward towards Europe. Although the Gulf Stream does not actually reach the shores of Europe, it nevertheless distributes its heated waters, under the influence of the prevailing south-west winds to the European waters, as is proved by our finding on the western shores of Ireland and on the coast of Norway the fruits of trees that grow in the hot zone of America; the west and south-west winds remain, therefore, long in contact with a sea water, whose temperature between 45 and 50 degrees of latitude, does not, even in January, sink below from 51 to 48° F. Northern Europe is thus separated by the influence of the Gulf Stream from the circle of polar ice by means of a sea free from ice; even at the coldest season of the year the limits of polar ice do not reach the European shores."

We are no very great sticklers for names; but we cannot but view it as a defect of the work, that in speaking of Electricity, the name of Franklin is not so much as mentioned as the discoverer of the positive and negative theory.

Dufay's is, perhaps, more generally, at the present day, adopted; but the hypothesis of Franklin is more simple, and explains equally satisfactorily the phenomena of electrical action.

The number and beauty of the wood-cuts struck us at once. They are unsurpassed in distinctness of outline and clearness of delineation.

We observe at page 28 the following: "The wood-cuts of the original German edition of this work, from which those in our present translation are copied, were impressed by copper type of this description," (i. e., by the galvano-plastic process.)

This volume, we learn, is the first of a series of works on different branches of science, to be published in London by Baillière, and republished in this country by the enterprising American house.

We sincerely wish success to the undertaking, believing, when finished, that the whole series will form a valuable scientific library.

